# Plasma miRNA expression profile in pediatric pineal pure germinomas

**DOI:** 10.3389/fonc.2024.1219796

**Published:** 2024-04-11

**Authors:** Mona Fakhry, Moatasem Elayadi, Mariam G. Elzayat, Omar Samir, Eslam Maher, Hala Taha, Mohamed El-Beltagy, Amal Refaat, Manal Zamzam, Mohamed S. Abdelbaki, Ahmed A. Sayed, Mark Kieran, Alaa Elhaddad

**Affiliations:** ^1^ Department of Pediatric Oncology, Children’s Cancer Hospital Egypt (CCHE-57357), Cairo, Egypt; ^2^ Department of Pediatric Oncology, National Cancer Institute (NCI), Cairo University, Cairo, Egypt; ^3^ Genomics and Epigenomics Program, Research Department, Children’s Cancer Hospital Egypt (CCHE-57357), Cairo, Egypt; ^4^ Clinical Research Department, Children’s Cancer Hospital Egypt (CCHE-57357), Cairo, Egypt; ^5^ Department of Pathology, National Cancer Institute (NCI), Cairo University and Children Cancer Hospital (CCHE-57357), Cairo, Egypt; ^6^ Department of Neurosurgery, Children’s Cancer Hospital Egypt (CCHE-57357) and Faculty of Medicine, Cairo University, Cairo, Egypt; ^7^ Radio-Diagnosis Department, National Cancer Institute (NCI), Cairo University and Children Cancer Hospital (CCHE-57357), Cairo, Egypt; ^8^ The Division of Hematology and Oncology, St. Louis Children’s Hospital, Washington University School of Medicine in St. Louis, St. Louis, MO, United States; ^9^ Faculty of Science, Ain Shams University, Cairo, Egypt

**Keywords:** germinoma, miRNA, liquid biopsy, plasma biomarkers, pineal body tumors

## Abstract

**Background:**

Pure germinomas account for 40% of pineal tumors and are characterized by the lack of appreciable tumor markers, thus requiring a tumor biopsy for diagnosis. MicroRNAs (miRNA) have emerged as potential non-invasive biomarkers for germ cell tumors and may facilitate the non-invasive diagnosis of pure pineal germinomas.

**Material and methods:**

A retrospective chart review was performed on all patients treated at the Children’s Cancer Hospital Egypt diagnosed with a pineal region tumor between June 2013 and March 2021 for whom a research blood sample was available. Plasma samples were profiled for miRNA expression, and DESeq2 was used to compare between pure germinoma and other tumor types. Differentially expressed miRNAs were identified. The area under the curve of the receive;r operating characteristic curve was constructed to evaluate diagnostic performance.

**Results:**

Samples from 39 pediatric patients were available consisting of 12 pure germinomas and 27 pineal region tumors of other pathologies, including pineal origin tumors [*n* = 17; pineoblastoma (*n* = 13) and pineal parenchymal tumors of intermediate differentiation (*n* = 4)] and others [*n* = 10; low-grade glioma (*n* = 6) and atypical teratoid rhabdoid tumor (*n* = 4)]. Using an adjusted *p*-value <0.05, three miRNAs showed differential expression (miR-143-3p, miR-320c, miR-320d; adjusted *p* = 0.0058, *p* = 0.0478, and *p* = 0.0366, respectively) and good discriminatory power between the two groups (AUC 90.7%, *p* < 0.001) with a sensitivity of 25% and a specificity of 100%.

**Conclusion:**

Our results suggest that a three-plasma miRNA signature has the potential to non-invasively identify pineal body pure germinomas which may allow selected patients to avoid the potential surgical complications.

## 1 Introduction

Pineal region tumors constitute 3%–8% of central nervous system (CNS) tumors in children (1). They include germ cell tumors (GCTs), pineal parenchymal tumors, and tumors arising from adjacent anatomical structures (2). GCTs constitute approximately 35% of all pineal region tumors and are typically divided into pure germinomas and non-germinomatous germ cell tumors (NGGCTs). The latter group constitutes a number of different entities including choriocarcinoma, yolk sack tumors, immature teratomas, and mixed histologies (3). GCTs are presumed to arise from mutated primordial germ cells of genital ridge origin or dysfunctional totipotent embryonic cells. Histologically, germinoma is the most undifferentiated GCT and is composed of undifferentiated large cells that resemble primordial germinal elements (4).

In practice, the diagnosis of pineal region neoplasms is based on clinical presentation, imaging, and histopathologic analysis (5). While magnetic resonance imaging is sensitive and critical for the assessment of metastatic disease as well as metachronous germinoma concurrently in the pineal and suprasellar regions, still the radiographic characteristics are very similar in all GCTs, therefore limiting its usefulness in determining the exact histology of these tumors. Germinomas can enhance diffusely and NGGCTs commonly may have associated hemorrhage, causing a more heterogeneous pattern of enhancement (3). In addition, reduced apparent diffusion coefficient (ADC) values, bithalamic extension, and thick peritumoral edema are significant features that are more frequent in germinomas than in NGGCTs (6). Radiological findings coupled with positive tumor markers can aid in the diagnosis of approximately 12% of GCT cases (7), and thus, surgical biopsy remains the current standard in diagnosing non-hormone secreting pineal-based lesions (8).

Serum and cerebrospinal fluid (CSF) biomarkers complement the standard diagnostic techniques by providing additional data before invasive procedures are performed (5). If possible, tumor markers [i.e., alpha-fetoprotein (AFP) and β-human chorionic gonadotropin (β-HCG)] should be detected in both the serum and cerebrospinal fluid (9). The clinical basis for identifying a secreting tumor is AFP >10 IU/L or β-HCG >50 IU/L. In germinomas, the levels of these tumor markers are not typically elevated; however, the β-HCG level is increased in a small number of germinomas because of its syncytiotrophoblast element, and still, it does not exceed the threshold of 50 IU/L. In this setting, some investigators would consider a slightly elevated β-HCG <50 IU/L in the context of typical imaging for a germinoma sufficiently characterized to avoid biopsy. Venkatasai et al. used an upper limit for β-HCG of 100 IU/L to differentiate germinoma from NGGCT, and interestingly, they considered that patients with β-HCG <20 IU/L are indicated for biopsy, while those with levels of 20–100 IU/L, a biopsy can be avoided (10). Mature teratomas do not secrete β-HCG or AFP. Embryonal carcinomas secrete a trace amount of β-HCG and AFP; however, these concentrations do not reach the diagnostic threshold (11). Hence, the value of these biomarkers for diagnosis and follow-up is restricted to specific malignant GCT subtypes as the levels are mostly elevated in tumors containing yolk sac tumor (YST) and choriocarcinoma (CHC), which collectively represent approximately 12% of all GCT cases (7, 12). Furthermore, both AFP and β-HCG can be elevated in other non-malignant conditions (13).

Open surgical resection and stereotactic or endoscopic biopsy are thus needed to confirm the diagnosis of marker-negative pineal region tumors (14). However, these procedures are associated with a higher risk of hemorrhage due to the presence of critical vascular structures in this area as well as potential damage to adjacent neural structures (15). Hence, a highly specific body fluid biomarker that offers a high degree of specificity for pure germinomas may permit a less invasive method of diagnosis as well as a potential marker for response assessment.

MicroRNA (miRNAs) are short non-coding RNAs that regulate gene expression and have been shown to be well-preserved in a range of specimen types including blood plasma and serum. miRNA expression profiling has helped to identify miRNAs that regulate a range of processes (16), and this has led to a considerable interest in them as biomarkers for cancer (17). Specific cancer-derived miRNAs that are detectable in the serum and plasma have been used as non-invasive biomarkers in many malignant conditions (18).

Previous studies of miRNA expression in GCT identified eight major miRNAs from the miR-371–373 and miR-302/367 clusters that were overexpressed in malignant GCT (19). Furthermore, certain miRNAs can help distinguish GCT from other brain tumors using CSF and serum samples (20). Moreover, in a global miRNA expression analysis of 12 tumor specimens from primary pediatric GCT of the brain, pure germinoma-specific upregulation of miR-142-5p and miR-146a-5p was reported, in contrast to overexpression of miR-335-5p and miR-654-3p in NGGCT (4). Yet, it is still unknown whether the detection of these miRNAs is discriminatory in the serum/plasma of pediatric CNS germinoma patients (21). We sought to identify plasma signatures that could distinguish pure germinomas of the pineal region from all other tumors in this area, thus potentially avoiding the need for surgical intervention in this very vulnerable part of the brain.

## 2 Materials and methods

This study included pediatric patients with pathologically proven pineal region tumors at the Children’s Cancer Hospital Egypt 57357 (CCHE 57357) between June 2013 and March 2021 for whom a blood sample was available in the research tissue bank. Patient charts were reviewed and relevant data were extracted. Patient-level data were maintained in a separate database, and linkage to the pathology and research blood samples was maintained only until data abstraction was completed.

All blood samples were prospectively collected at the initial presentation and before the initiation of therapy on an Institutional Review Board (IRB)-approved tissue collection registry protocol with parental consent that allowed for future research use of the material.

### 2.1 Ethical and IRB approval

All procedures performed in the study involving human participants were in accordance with the ethical standards of the IRB of CCHE 57357 and with the 1964 Declaration of Helsinki and its later amendments or comparable ethical standards. This study was granted a waiver for additional consent by the IRB since all information collected was available by retrospective review of both the chart and tissue.

### 2.2 Sample collection and RNA isolation

All plasma samples were retrieved from the CCHE 57357 biorepository. Total RNA, including miRNA, was extracted from 250 μl of plasma using the miRNeasy Mini Kit as per the manufacturer’s instructions (Qiagen, Germany). RNA concentration was determined using the Qubit RNA HS Assay Kit (Invitrogen, USA).

### 2.3 MiSeq library preparation and sequencing

To generate the miRNA library, samples were adjusted to include 250 ng of pure RNA in 10.5 µl. RNA was converted to a DNA library using the NEXTFLEX® Small RNA-Seq Kit (PerkinElmer Inc., MA, USA). In brief, RNA ligation was performed using the manufacturer’s recommended 3′ and 5′ ligation solution mix. Ligated RNA was reverse-transcribed to generate cDNA, which was then amplified using a PCR master mix with a unique primer for each library. The band of interest was trimmed and recovered in 300 μl of elution buffer using 10% TBE-PAGE. Bioanalyzer DNA assay (Agilent, CA, USA) was used to determine the size distribution of the library, and the concentration was determined using Qubit dsDNA HS Assay (Thermo Fisher Scientific, MA, USA). All 47 sample libraries were pooled and sequenced on an Illumina MiSeq instrument using the MiSeq Reagent Kit v3 (150 cycles) (Illumina Inc., San Diego, CA, USA).

### 2.4 Bioinformatics data analysis

Data analysis was performed using Unix-based tools (**Supplementary Figure 1**). The quality of raw reads was evaluated using FastQC (22). Cutadapt (23) was used to trim the following adaptor sequence “TGGAATTCTCGGGTGCCAAGG” followed by removing four bases from both read ends, as suggested by the manufacturer. Processed reads were filtered in which only reads between 15 and 28 nucleotides were retained. The quality of filtered reads was inspected using FastQC and the output was summarized using MultiQC (24).

Filtered reads were aligned using Bowtie 1 (25) against human genome reference GRCh38 downloaded from the NCBI database (accession number GCA_000001405.29) using default parameters with no mismatch option enabled. Mapped reads were quantified using featureCounts (26) and miRNA coordination retrieved from the miRBase database (27). Principal component analysis (PCA) was performed using the pca function.

Differential expression (DE) was performed on generated counts using DESeq2 (28) package in R V4.1.2. Data were normalized and DESeq2 was used to compare between pure germinoma samples and other tumor subtypes. Two sets of differentially expressed miRNAs were selected, one with a log2 fold change of 1.5 and an adjusted *p*-value of 0.5 and one with an adjusted *p*-value of 0.05. Functional enrichment was performed on a selected miRNA list using MIENTURNET (29).

### 2.5 Statistical analysis

The diagnostic potential for the differentially expressed miRNAs was determined using receiver operating characteristics (ROC) analysis for the regularized log transformation of raw counts, and their discriminatory power was investigated via the area under the curve (AUC). Predicted probabilities from binary logistic regression were calculated for analyzing the miRNAs combined. IBM SPSS Statistics version 20 and pROC v1.18.0 (30) were used.

## 3 Results

### 3.1 Patient characteristics

The cohort was comprised of pure germinomas (*n* = 15), pineal origin tumors [*n* = 19; pineoblastoma (*n* = 14) and low-grade pineal parenchymal tumors of intermediate differentiation (PPTIDs) (*n* = 5)], and other tumors [*n* = 13; low-grade glioma (LGG) (*n* = 9) and atypical teratoid rhabdoid tumor (AT/RT) (*n* = 4)]. Pineal region NGGCTs with elevation of serum and/or CSF β-HCG >50 IU/L or elevation of serum and/or CSF AFP >10 IU/L were excluded from the cohort as these tumors were diagnosed without a biopsy. β-HCG and AFP levels in the serum and CSF of patients with pure germinoma are listed in **Table 1**.

**Table 1:** β-HCG and AFP values in the serum and CSF of patients with pure germinoma.

Pure germinoma samples	Serum markers		CSF markers	
	β-HCG (IU/L)	AFP (ng/ml)	β-HCG (IU/L)	AFP (ng/ml)
**1**	0.3	2	11.8	0
**2**	<1.2	3	3	0.17
**3**	<1.2	5	1.28	0.02
**4**	<1.2	1	16.00	0.05
**5**	4.9	2	11	0.13
**6**	<1.2	2	<1.2	0
**7**	<1	1	NA	NA
**8**	<1	9	2.74	0.25
**9**	<1	NA	<1.2	0
**10**	<1	1	1.4	0
**11**	<1	1	1.09	0.01
**12**	7.1	0.64	2	0

NA, not available.

The median age for the entire cohort was 11 years (range, 1–16 years), consisting of 32 (68.1%) male and 15 (31.9%) female patients.

### 3.2 Identification of differentially expressed miRNA

**Figure 1:**
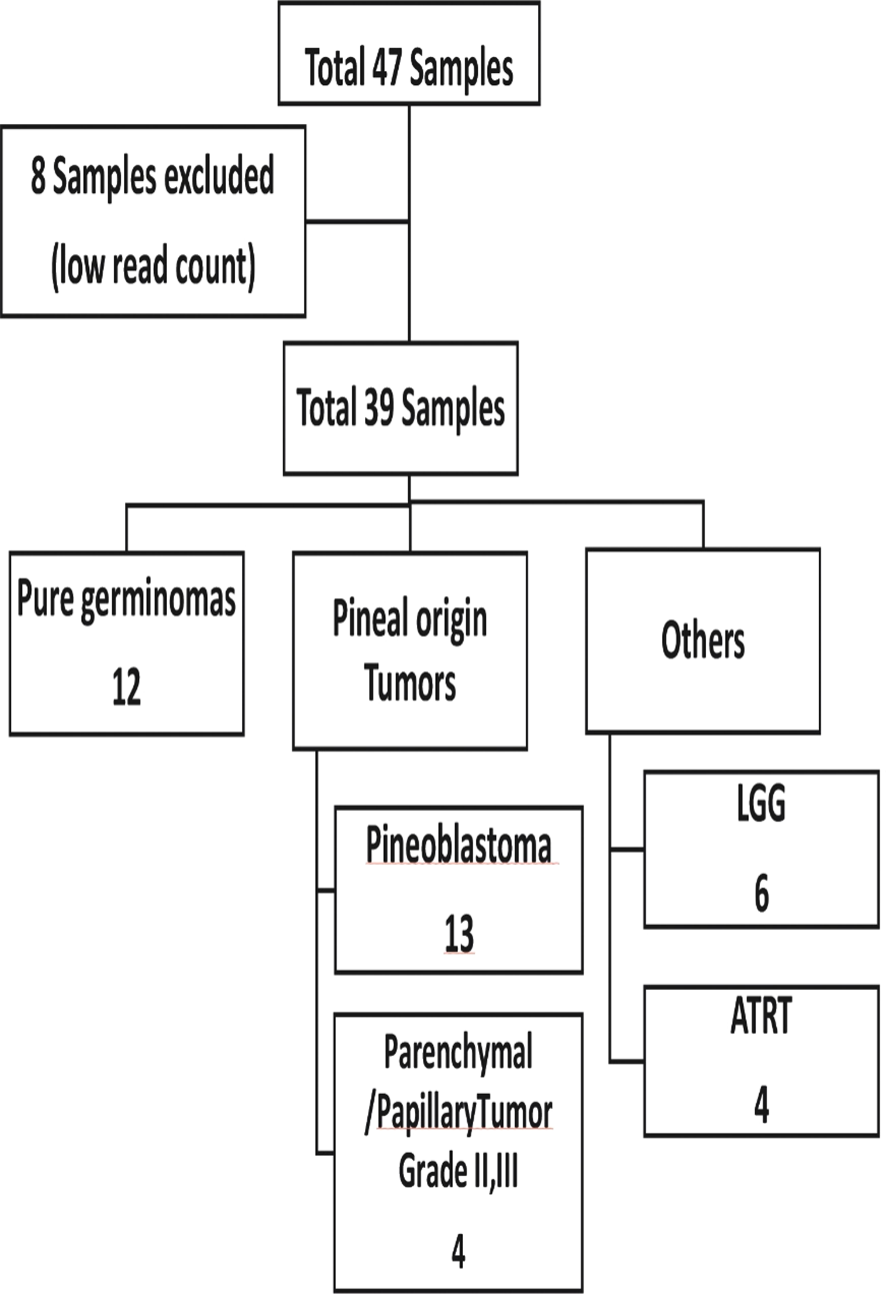
Sample distribution according to initial patient diagnosis.

A total of 47 plasma samples were profiled for the expression of global miRNAs. Eight samples were excluded due to low read counts (<100,000 reads), and the remaining 39 samples were used for further analysis (**Figure 1**).

PCA was performed to investigate the differences between sample groups. The variation between samples was 87.9% in PC1 and 6.07% in PC2. There was no clear separation between pure germinoma miRNA plasma profile and the other group of pineal tumors (**Supplementary Figure 2**).

**Figure 2:**
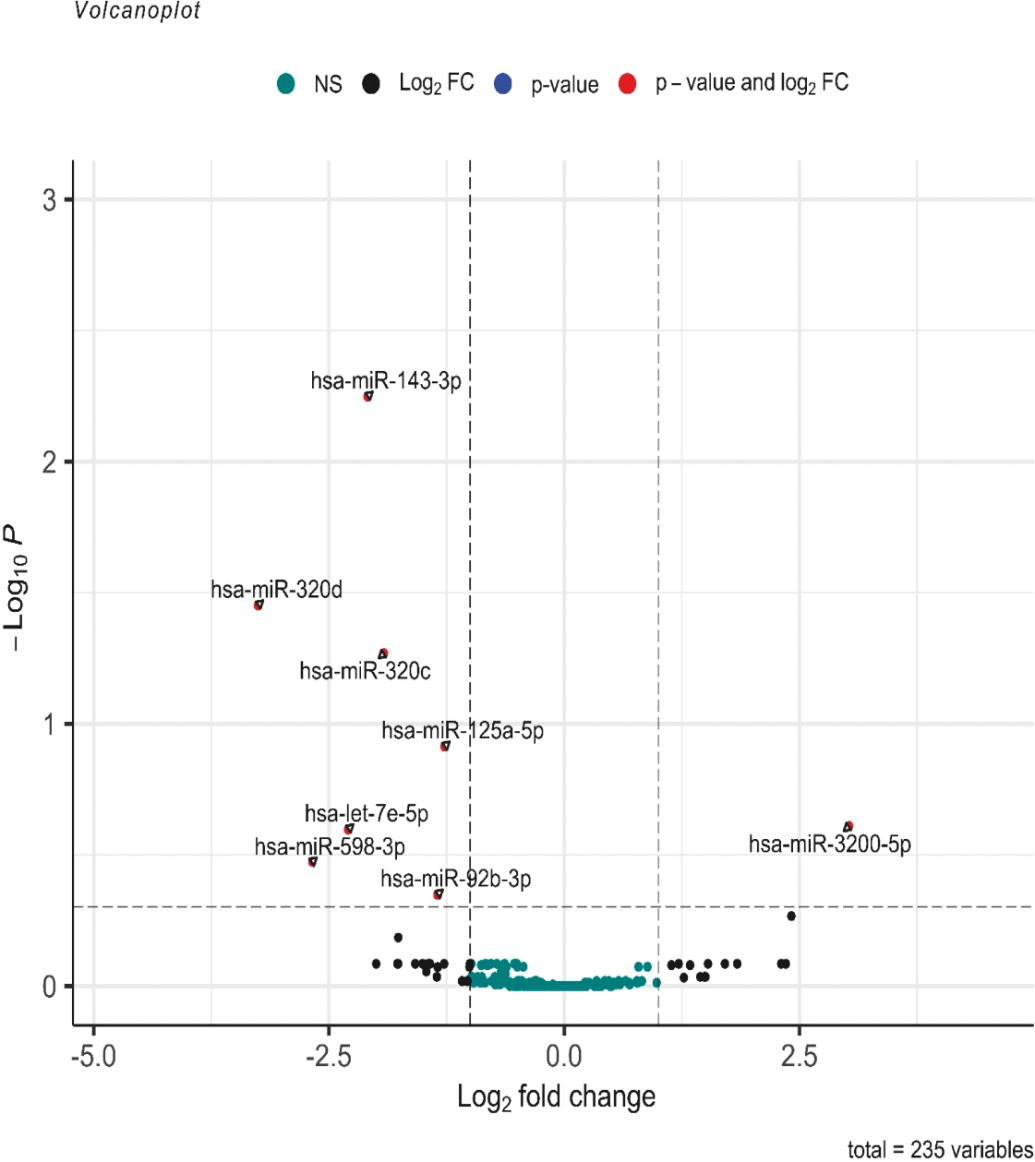
Volcano plot of differentially expressed miRNAs. log2 (fold change) is plotted against −log10 (*p*-value), where the *p*-value is from the differential miRNA expression test. The vertical dash line represents a fold change of 1, while the horizontal dash line represents the *p*-value threshold. Adjusted *p*-value cutoff <0.5. The red dots denote miRNAs that meet both the FC ≥1 and adjusted *p*-value <0.5 criteria, while black dots meet FC ≥1 but not the adjusted *p*-value <0.5 criteria. Green dots did not meet either criteria.

Among the 2,652 miRNA genes quantified, 235 genes had count per million reads (cpm) greater than 0.25 in at least 60% of the samples and were used for differential gene expression analysis. When comparing the 12 pure germinoma samples and 27 other pineal region tumors, eight of the 235 miRNAs were differentially expressed, using an adjusted *p*-value <0.5 (volcano plot in **Figure 2**). Of the eight differentially expressed miRNAs, seven were downregulated and only one (miRNA-3200-5p) was upregulated.

Further analysis between the two groups was performed using a more stringent *p*-value. miRNAs with adjusted *p*-values <0.05 were considered statistically significant and differentially expressed. Three of the eight miRNAs showed differential expression (miR-143-3p, miR-320c, and miR-320d with adjusted *p* = 0.0058, *p* = 0.0478, and *p* = 0.0366, respectively) (see volcano plot in **Figure 2**).

### 3.3 Pathway enrichment analysis of downregulated miRNAs in pure germinomas

**Figure 3:**
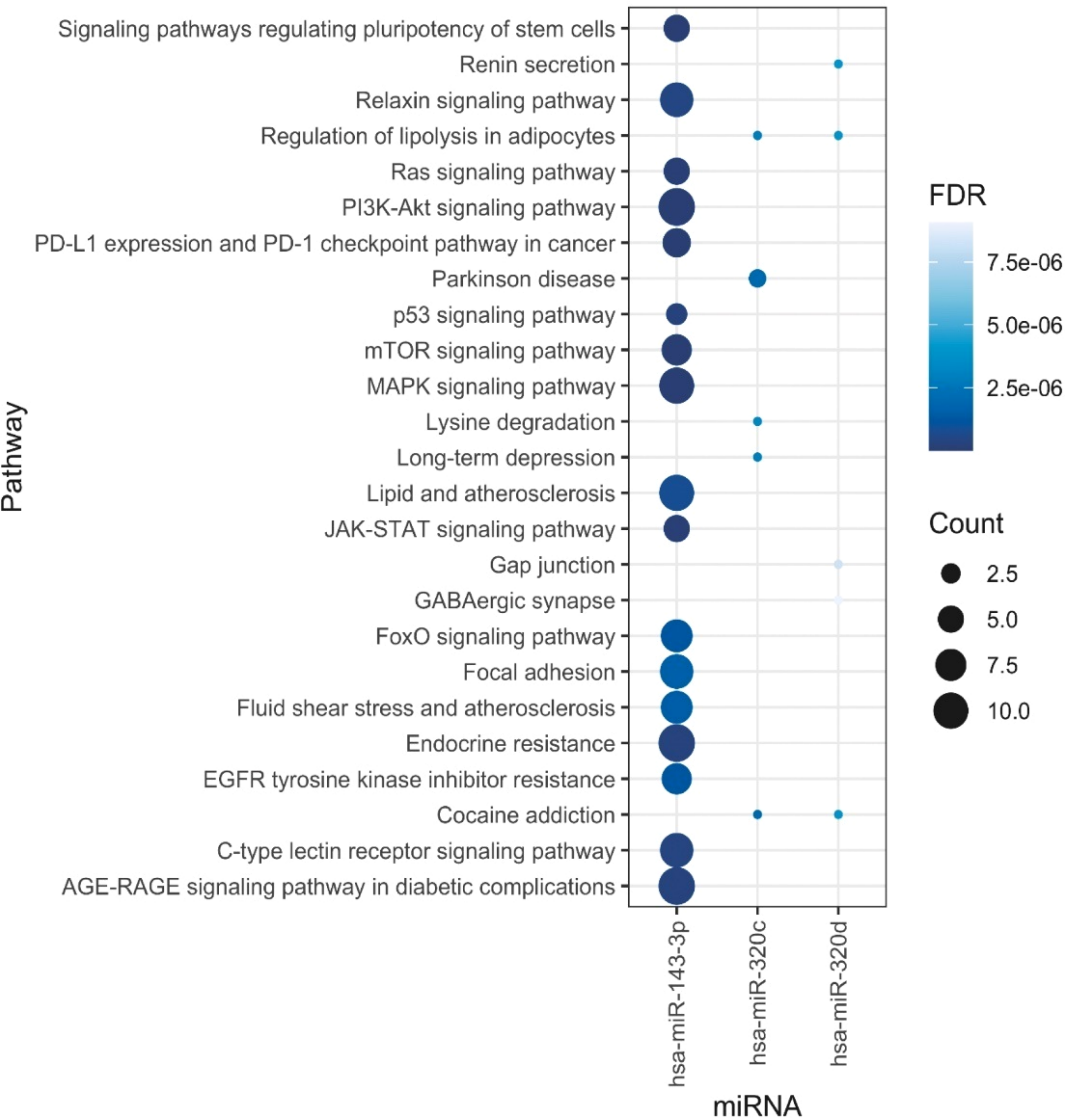
Network of genes associated with differentially expressed miRNAs.

Functional annotation of the three differentially expressed miRNAs combined revealed 337 regulated genes by those miRNAs in miRTarBase (31), an interaction network represented in **Figure 3**.

**Figure 4:**
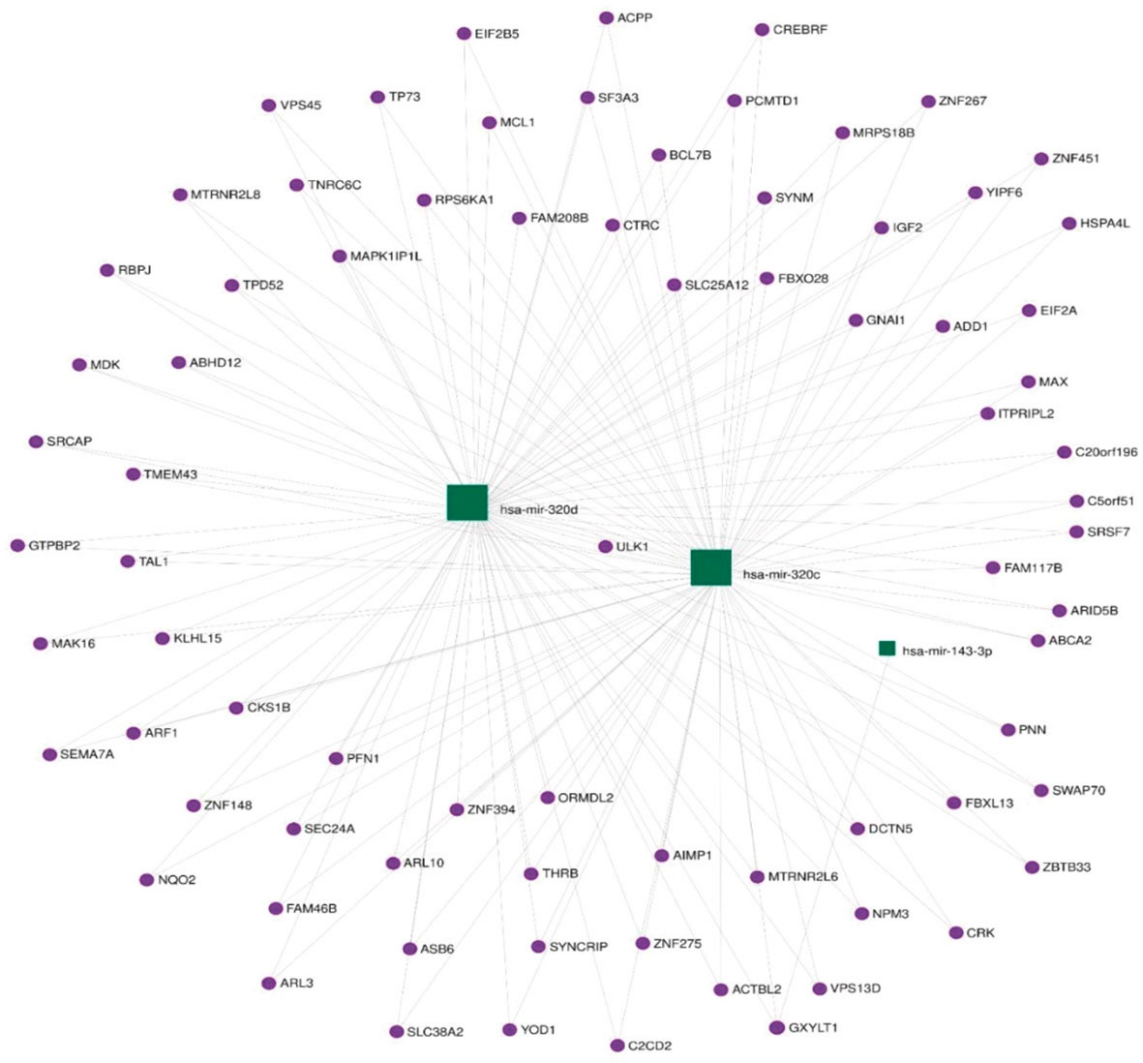
Top enriched pathways representing the number of regulated genes per miRNA.

The KEGG database (32) was searched using retrieved genes and miRNAs, and 406 different pathways were enriched. The miR-143-3p was found to regulate a number of genes that are involved in important pathways known to be linked to GCT including the phosphoinositide-3-kinase/Akt (PI3K–Akt) signaling pathway, mitogen-activated protein kinase (MAPK) signaling pathway, mammalian target of rapamycin (mTOR) signaling pathway, and others. The top enriched pathways are presented in **Figure 4**.

### 3.4 ROC analysis for miRNA in pineal tumors

**Figure 5:**
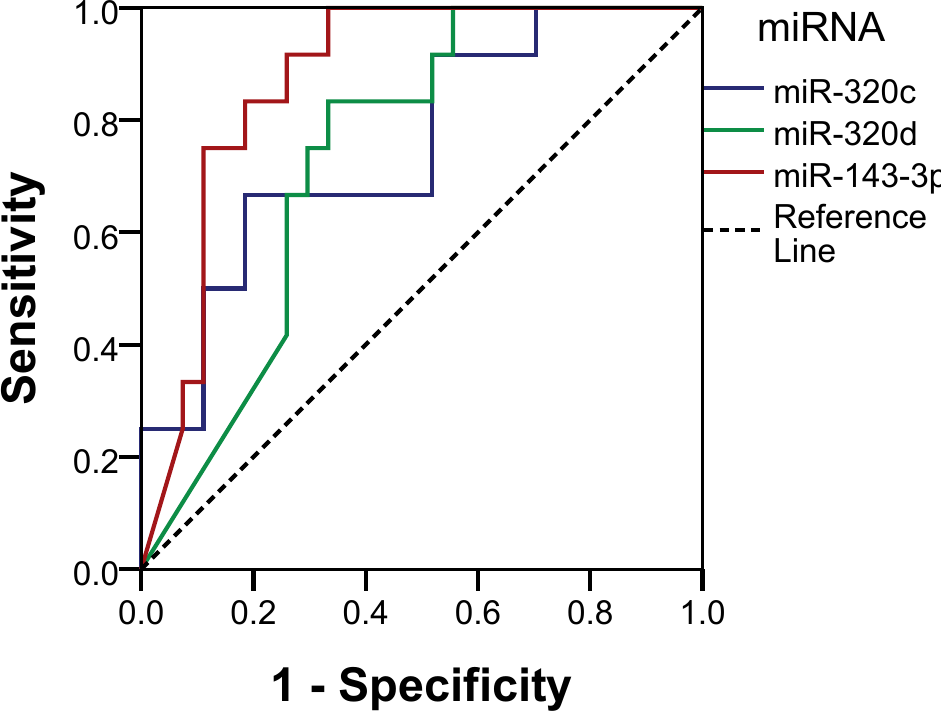
Receiver operating characteristic (ROC) curve analysis of individual miRNAs.

Using normalized counts, the diagnostic potential for the miRNAs was analyzed using ROC curve analysis. All three miRNAs showed good discriminatory power, as shown by their AUC: miR-143-3p (AUC 87.3%; sensitivity 100%, specificity 66.7%, *p* < 0.001), miR-320c (AUC 75.3%; sensitivity 66.7%, specificity 81.5%, *p* = 0.013), and miR-320d (AUC 73.9%; sensitivity 83.3%, specificity 66.7%, *p* = 0.018) (**Figure 5**). We established a cutoff point for the normalized counts (regularized log transformation of the raw counts). The ROC curve coordinates for the three miRNAs provided corresponded to the following cutoffs: miR_320c: 27.55, miR_143_3p: 33.18, and miR_320d: 6.91.

**Figure 6:**
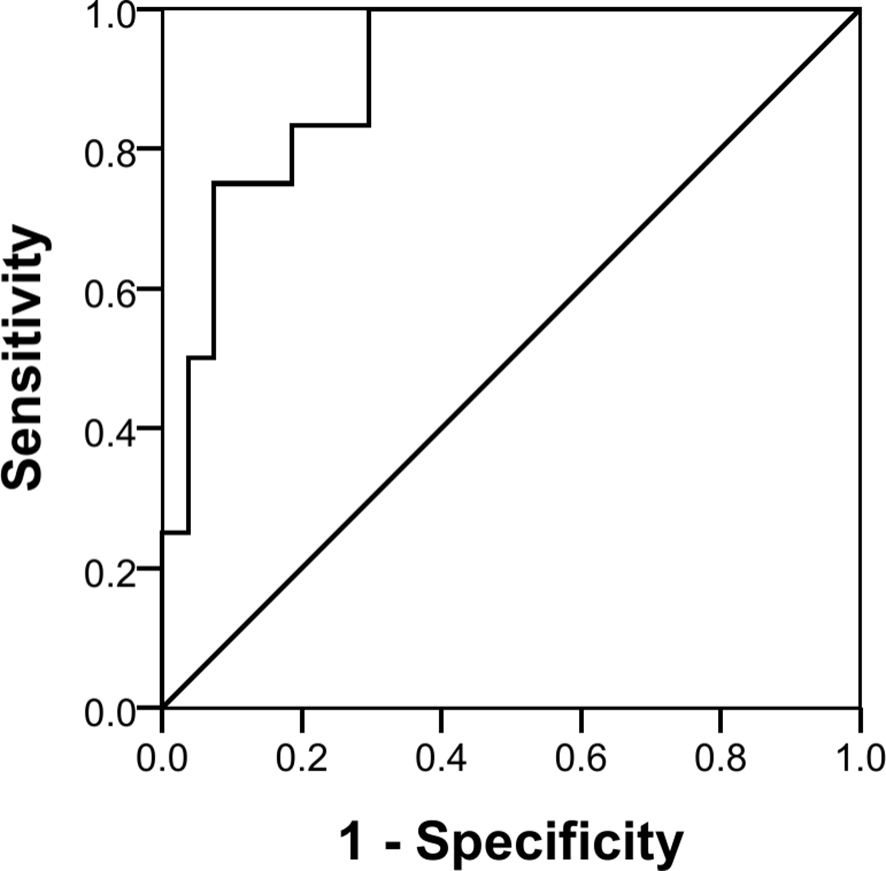
Receiver operating characteristic (ROC) curve analysis of three miRNAs combined.

The three miRNAs combined also showed a good discriminatory power (AUC 90.7%, *p* < 0.001) with a sensitivity of 25% and a specificity of 100% in identifying pure germinomas from other pineal region tumors. Using the same coordinates, predictive values were calculated showing a positive predictive value (PPV) of 100% and a negative predictive value (NPV) of 75% (**Figure 6**).

## 4 Discussion

The most common pineal region neoplasms are GCTs, and they are divided into pure germinomas (55%–65%) and NGGCTs (35%–45%) (33). Pure germinomas, which often occur in male adolescent patients, account for 40% of pineal region neoplasms (34).

Pure germinomas do not secrete appreciable tumor markers, and thus, a tumor biopsy is typically required for diagnosis (3). Such procedures carry risks of morbidity due to difficulties in surgical access to the pineal region (20). The surgical options for biopsy are stereotactic, endoscopic, or open microsurgical approach, while only the latter two options enable concomitant hydrocephalus treatment (35). The risk of lasting morbidity from open resection is 3% to 10%, while mortality rates range from 4% to 10%. Meanwhile, the mortality and morbidity rates for stereotactic biopsy of the pineal region range between 1% and 1.3% (36). Finding a blood and/or CSF biomarker for pure germinomas would offer many benefits, and circulating microRNAs hold great clinical promise in this area (37). It has been previously shown that pure germinomas and NGGCTs (intra- and extracranial) are characterized by high expression levels of all eight microRNA members of the miR-371~373 and miR-302/367 clusters in the serum and CSF, regardless of patient age, tumor site, or histological subtype (19).

This biomarker study presents the results of an miRNA profile expression analysis of pineal region tumors aiming to identify potential biomarkers in plasma that can be used to diagnose pineal body pure germinoma and differentiate them from other pineal region tumors identified through imaging without the need for a tissue biopsy.

CSF is a suitable repository of clinical biomarkers for CNS tumors, and an increasing number of studies have reported that CSF-derived biomarkers are more abundant than those in the peripheral blood and other sources (38). Qu et al. have also shown that miR-21 level in the CSF enabled the identification of glioma patients with higher sensitivity and specificity in comparison with the plasma/serum miR-21 level (39). Due to the unavailability of CSF samples at diagnosis, analysis of miRNAs in the CSF was not included in our study.

Several studies have reported both plasma and serum as acceptable specimens for circulating miRNA analysis (40). Although some studies have shown no or minimal difference between serum and plasma (41), others noted that plasma samples provide a higher recovery of miRNA compared with serum (42). For that reason, we chose plasma over serum for the conduction of our analysis.

Due to the low reproducibility in the quantification of circulating miRNAs in plasma compared with CSF (43), we used a permissive approach in our analysis using a less stringent *p*-value (*p* < 0.5), where we identified eight miRNAs that were differentially expressed when comparing the pure germinoma group with the other group of pineal region tumors. We then applied a more stringent *p*-value (*p* < 0.05) in order to discover a more specific expression profile, where only three of the eight miRNAs showed significant differential expression.

While the results presented need to be confirmed and expanded, the finding that the three combined miRNAs demonstrated good discriminatory power (AUC 90.7%, *p* < 0.001) in a subset of patients (sensitivity of 25%) with a pineal-based pure germinoma [100% specificity with a false discovery rate (FDR) of 0%] suggests that a subset of patients might be able to avoid a costly and potentially morbid surgical procedure.

In our samples, we were unable to detect the previously documented miR-371~373 and miR-302/367 clusters (20). This is in contrast to a recent study by Schönberger et al., where they demonstrated the suitability of miR-371a, miR-372, miR-367, and miR-302d in serum and CSF for the diagnosis of CNS GCT, particularly in biomarker-negative pure germinoma (21). This discrepancy can be attributed to the difference in methodology between the two studies. While we opted to use unselected material as the starting point, they preamplified RT-qPCR involving only these specific miRNAs. This makes it difficult to discover if other miRNAs from different clusters would have been differentially expressed in their cohort. Moreover, they used healthy subjects as controls, unlike our study where samples from patients with other pineal region tumors were used for comparison. This is an important difference as the primary goal of this work was to investigate if miRNAs can be used to separate patients with pure germinomas from all of the other tumors of the pineal region in pediatric patients as a method to avoid unnecessary intervention.

Another possible explanation for the low expression levels of these miRNA clusters in our samples is the possibility that these clusters were not efficiently labeled, sequenced, or amplified and thus lost to analysis. Several studies have shown that cellular contamination and hemolysis of samples can be a major cause of variation in miRNA levels not related to any biological difference (44).

A review of the miRNAs from the pure germinoma group identified a number of functions associated with signal transduction, cell cycle, development, and morphogenesis. miR-143-3p, for example, is known to regulate pathways that mediate cellular processes important in oncogenesis and malignant progression including the MAPK, mTOR, and PI3K–Akt signaling pathways in addition to others (32). The PI3K/AKT and MAPK pathways have been implicated in the pathogenesis of pure germinoma as they are both present in approximately 83% of tumor cells (45). MAPK pathway alterations are more frequent in pure germinomas than NGGCTs and have a tendency to correlate with a better outcome compared with PI3K pathway mutations. Upregulation of the MAPK pathway by somatic point mutations represents the dominant genetic alteration in pure germinomas (64.3% of cases) (46). Further investigation on the possible functional significance of the miR-143-3p and the other miRNAs is needed.

Our study has a number of important limitations. The main limitation is linked to the small number of cases, which is expected with such a rare tumor. To address this issue, multicenter or consortium-based studies will be needed. Since only a blood sample is required, such a study would be feasible and is currently under consideration. Another limitation is the absence of CSF samples, which may have a much better signal-to-noise ratio, as has been identified in the detection of *BRAF* and *H3K27M* mutations (47). In addition, given the unique focus of our research on pineal body tumors, the validation of our findings using existing publicly available data was not feasible due to the lack of comparable datasets. Finally, due to the rarity of this patient population, a long duration of sample collection was required, which could impact the quality of the starting material from blood.

In conclusion, the results of the present study suggest that a three-plasma miRNA signature has the potential to serve as a diagnostic biomarker panel for pineal body pure germinomas. While currently only of potential value to a small proportion of pineal-based tumor cases, this approach may open up the opportunity to expand these results and generate hypotheses for future investigations. Prospective testing of these markers on larger samples is warranted in the hopes that avoidance of surgical biopsy can be achieved for patients with tumors in this critical area of the brain. Coupling plasma/serum with CSF samples and validation with quantitative real-time PCR (qPCR) for these miRNAs alongside with miR-371~373 and miR-302/367 clusters are recommended in future studies.

## Data availability statement

The datasets generated and analyzed during the current study are published online on NCBI with BioProject ID PRJNA880483 https://www.ncbi.nlm.nih.gov/sra/PRJNA880483.

## Ethics statement

This study was approved by the Scientific Advisory Committee (SMAC), and the Institutional Review Board (IRB) approved the study that was granted on 11 November 2019 with serial number 57/2019. The studies were conducted in accordance with the local legislation and institutional requirements. Written informed consent for participation in this study was provided by the participants’ legal guardians/next of kin.

## Author contributions

MA, MF, MoE, MK, AS, MZ, and ME-B contributed to the study conception and design. Data collection was performed by MF and EM, data revision was performed by MF, HT, and AR. Material preparation and data analysis were performed by MF, MaE, OS, and EM. The first draft of the manuscript was written by MF, MoE, MaE, OS and EM. MK, AE, MA, AS, and MZ contributed to critical revision of the article and commented on previous versions of the manuscript. All authors contributed to the article and approved the submitted version.

## References

[1] Dumrongpisutikul N Intrapiromkul J Yousem DM. Distinguishing between germinomas and pineal cell tumors on MR imaging. AJNR. (2012) 33:550–5. doi: 10.3174/AJNR.A2806 PMC7966430 22173760

[2] Favero G. Pineal gland tumors: A review. Cancers (Basel). (2021) 13:1547. doi: 10.3390/cancers13071547 33801639 PMC8036741

[3] Echevarría ME Fangusaro J Goldman S. Pediatric central nervous system germ cell tumors:A review. Oncologist. (2008) 13:690–9. doi: 10.1634/theoncologist.2008-0037 18586924

[4] Wang HW Wu YH Hsieh JY Liang ML Chao ME Liu DJ. Pediatric primary central nervous system germ cell tumors of different prognosis groups show characteristic miRNome traits and chromosome copy number variations. BMC Genomics. (2010) 11:132. doi: 10.1186/1471-2164-11-132 20178649 PMC2837036

[5] Carr C O’neill BE Hochhalter CB Strong MJ Ware ML. Biomarkers of pineal region tumors: A review. Ochsner J. (2019) 19:26–31. doi: 10.31486/TOJ.18.0110 30983898 PMC6447205

[6] Yang M Wang J Zhang L Liu J. (2023). Update on MRI in pediatric intracranial germ cell tumors-The clinical and radiological features. Frontiers in pediatrics 11:1141397. doi: 10.3389/fped.2023.1141397 37215600 PMC10192609

[7] Mufti ST Jamal A. Primary intracranial germ cell tumors. Asian J Neurosurg. (2012) 7:197–202. doi: 10.4103/1793-5482.106652 23559987 PMC3613642

[8] Solomou AG. Magnetic resonance imaging of pineal tumors and drop metastases: A review approach. Rare Tumors. (2017) 9:69–76. doi: 10.4081/rt.2017.6715 PMC5661140 29142658

[9] Fujimaki T Mishima K Asai A Tabuchi K Kobayashi M Suzuki I. Levels of β-human chorionic gonadotropin in cerebrospinal fluid of patients with Malignant germ cell tumor can be used to detect early recurrence and monitor the response to treatment. Jpn J Clin Oncol. (2000) 30:291–4. doi: 10.1093/jjco/hyd076 11007160

[10] Venkatasai J Balakrishnan R Rajkrishna B Sebastain P John RR Vanjare HA. A pragmatic diagnostic approach to primary intracranial germ cell tumors and their treatment outcomes. CNS Oncol. (2021) 10:CNS79. doi: 10.2217/cns-2021-0012 34806399 PMC8610002

[11] Kong Z Wang Y Dai C Yao Y Ma W Wang Y. Central nervous system germ cell tumors: A review of the literature. J Child Neurol. (2018) 33:610–20. doi: 10.1177/0883073818772470 29848146

[12] Murray MJ Nicholson JC. α-fetoprotein. Archives of disease in childhood. Educ Pract Edition. (2011) 96:141–7. doi: 10.1136/ADC.2011.213181 21613305

[13] Murray MJ Nicholson JC Coleman N. Biology of childhood germ cell tumours, focussing on the significance of microRNAs. Andrology. (2015) 3:129–39. doi: 10.1111/ANDR.277 PMC4409859 25303610

[14] Mathieu D Iorio-Morin C. Stereotactic radiosurgery for pineal region tumors. Prog Neurological Surgery. (2019) 34:173–83. doi: 10.1159/000493062 31096261

[15] Cheng G Yu X Zhao H Cao W Li H Li Q. Complications of stereotactic biopsy of lesions in the sellar region, pineal gland, and brainstem: A retrospective, single-center study. Medicine. (2020) 99:e18572. doi: 10.1097/MD.0000000000018572 32080071 PMC7034708

[16] Pritchard CC Cheng HH Tewari M. MicroRNA profiling: approaches and considerations. Nat Rev Genet. (2012) 13:358–69. doi: 10.1038/NRG3198 PMC4517822 22510765

[17] Lu J Getz G Miska EA Alvarez-Saavedra E Lamb J Peck D. MicroRNA expression profiles classify human cancers. Nature. (2005) .435:834–8. doi: 10.1038/NATURE03702 15944708

[18] Boeri M Verri C Conte D Roz L Modena P Facchinetti F. MicroRNA signatures in tissues and plasma predict development and prognosis of computed tomography detected lung cancer. Proc Natl Acad Sci United States America. (2011) 108:3713–8. doi: 10.1073/PNAS.1100048108 PMC3048155 21300873

[19] Palmer RD Murray MJ Saini HK Van Dongen S Abreu-Goodger C Muralidhar B. Malignant germ cell tumors display common microRNA profiles resulting in global changes in expression of messenger RNA targets. Cancer Res. (2010) 70:2911–23. doi: 10.1158/0008-5472.CAN-09-3301 PMC3000593 20332240

[20] Murray MJ Bell E Raby KL Rijlaarsdam MA Gillis AJM Looijenga LHJ. A pipeline to quantify serum and cerebrospinal fluid microRNAs for diagnosis and detection of relapse in paediatric Malignant germ-cell tumours. Br J Cancer. (2016) 114:151–62. doi: 10.1038/bjc.2015.429 PMC4815809 26671749

[21] Schönberger S Mohseni MM Ellinger J Tran GVQ Becker M Claviez A. MicroRNA-profiling of miR-371~373- and miR-302/367-clusters in serum and cerebrospinal fluid identify patients with intracranial germ cell tumors. J Cancer Res Clin Oncol. (2022). doi: 10.1007/s00432-022-03915-4 PMC9931786 35171328

[22] Andrews S. FastQC: A Quality Control Tool for High Throughput Sequence Data (2010). Available online at: http://www.bioinformatics.babraham.ac.uk/projects/fastqc/ (Accessed 23 February 2022).

[23] Martin M. Cutadapt removes adapter sequences from high-throughput sequencing reads. EMBnet J. (2011) 17:10–2. doi: 10.14806/EJ.17.1.200

[24] Ewels P Magnusson M Lundin S Käller M. MultiQC: summarize analysis results for multiple tools and samples in a single report. Bioinformaticss. (2016) 32:3047–8. doi: 10.1093/BIOINFORMATICS/BTW354 PMC5039924 27312411

[25] Langmead B Trapnell C Pop M Salzberg SL. Ultrafast and memory-efficient alignment of short DNA sequences to the human genome. Genome Biol. (2009) 10:1–10. doi: 10.1186/gb-2009-10-3-r25 PMC2690996 19261174

[26] Liao Y Smyth GK Shi W. FeatureCounts: an efficient general purpose program for assigning sequence reads to genomic features. Bioinformatics. (2014) 30:923–30. doi: 10.1093/BIOINFORMATICS/BTT656 24227677

[27] Griffiths-Jones S Grocock RJ van Dongen S Bateman A Enright AJ. miRBase: microRNA sequences, targets and gene nomenclature. Nucleic Acids Res. (2006) 34(Database issue):D140–4. doi: 10.1093/NAR/GKJ112 PMC1347474 16381832

[28] Love MI Huber W Anders S. Moderated estimation of fold change and dispersion for RNA-seq data with DESeq2. Genome Biol. (2014) 15:1–21. doi: 10.1186/s13059-014-0550-8 PMC4302049 25516281

[29] Licursi V Conte F Fiscon G Paci P. MIENTURNET: An interactive web tool for microRNA-target enrichment and network-based analysis. BMC Bioinf. (2019) 20:1–10. doi: 10.1186/s12859-019-3105-x PMC6829817 31684860

[30] Robin X Turck N Hainard A Tiberti N Lisacek F Sanchez JC. pROC: An open-source package for R and S+ to analyze and compare ROC curves. BMC Bioinf. (2011) 12:1–8. doi: 10.1186/1471-2105-12-77/TABLES/3 PMC3068975 21414208

[31] Huang HY Lin YCD Li J Huang KY Shrestha S Hong HC. miRTarBase 2020: updates to the experimentally validated microRNA-target interaction database. Nucleic Acids Res. (2020) 48:D148–54. doi: 10.1093/NAR/GKZ896 PMC7145596 31647101

[32] Kanehisa M Goto S Furumichi M Tanabe M Hirakawa M. KEGG for representation and analysis of molecular networks involving diseases and drugs. Nucleic Acids Res. (2010) 38(Database issue):D355–60. doi: 10.1093/NAR/GKP896 PMC2808910 19880382

[33] Frappaz D Dhall G Murray MJ Goldman S Faure Conter C Allen J. EANO, SNO and Euracan consensus review on the current management and future development of intracranial germ cell tumors in adolescents and young adults. Neuro-Oncology. (2022) 24:516. doi: 10.1093/NEUONC/NOAB252 34724065 PMC8972311

[34] Smirniotopoulos JG Rushing E Mena H. Pineal region masses: differential diagnosis. Radiographics : A Rev Publ Radiological Soc North America Inc. (1992) 12:577–96. doi: 10.1148/RADIOGRAPHICS.12.3.1609147 1609147

[35] Schulz M Afshar-Bakshloo M Koch A.Copper D Driever PH Tietze A Grün A. Management of pineal region tumors in a pediatric case series. Neurosurg Rev. (2021) 44:1417–27. doi: 10.1007/s10143-020-01323-1 PMC8121748 32504201

[36] Shabo E Czech T Nicholson JC Mallucci C Mottolese C Piatelli G. Evaluation of the perioperative and postoperative course of surgery for pineal germinoma in the SIOP CNS GCT 96 trial. Cancers. (2022) 14:3555. doi: 10.3390/cancers14143555 35884617 PMC9323477

[37] Murray MJ Ajithkumar T Harris F Williams RM Jalloh I Cross J. Clinical utility of circulating miR-371a-3p for the management of patients with intracranial Malignant germ cell tumors. Neuro-Oncol Adv. (2020) 2:vdaa048. doi: 10.1093/NOAJNL/VDAA048 PMC7236383 32642701

[38] Xiao F Lv S Zong Z Wu L Tang X Kuang W. Cerebrospinal fluid biomarkers for brain tumor detection: clinical roles and current progress. Am J Trans Res. (2020) 12:1379. PMC7191171 32355549

[39] Qu K Lin T Pang Q Liu T Wang Z Tai M. Extracellular miRNA-21 as a novel biomarker in glioma: Evidence from meta-analysis, clinical validation and experimental investigations. Oncotarget. (2016) 7:33994–4010. doi: 10.18632/oncotarget.9188 PMC5085133 27166186

[40] Kroh EM Parkin RK Mitchell PS Tewari M. Analysis of circulating microRNA biomarkers in plasma and serum using quantitative reverse transcription-PCR (qRT-PCR). Methods (San Diego Calif.). (2010) 50:298–301. doi: 10.1016/J.YMETH.2010.01.032 20146939 PMC4186708

[41] Wang K Yuan Y Cho JH McClarty S Baxter D Galas DJ. Comparing the microRNA spectrum between serum and plasma. PloS One. (2012) 7:e41561. doi: 10.1371/JOURNAL.PONE.0041561 22859996 PMC3409228

[42] McDonald JS Milosevic D Reddi HV Grebe SK Algeciras-Schimnich A. Analysis of circulating microRNA: preanalytical and analytical challenges. Clin Chem. (2011) 57:833–40. doi: 10.1373/CLINCHEM.2010.157198 21487102

[43] Larrea E Sole C Manterola L Goicoechea I Armesto M Arestin M. New concepts in cancer biomarkers: circulating miRNAs in liquid biopsies. Int J Mol Sci. (2016) 17:627. doi: 10.3390/IJMS17050627 27128908 PMC4881453

[44] Blondal T Jensby Nielsen S Baker A Andreasen D Mouritzen P Wrang Teilum M. Assessing sample and miRNA profile quality in serum and plasma or other biofluids. Methods (San Diego Calif.). (2013) 59:S1–6. doi: 10.1016/J.YMETH.2012.09.015 23036329

[45] Schulte SL Waha A Steiger B Denkhaus D Dörner E Calaminus G. CNS germinomas are characterized by global demethylation, chromosomal instability and mutational activation of the Kit-, Ras/Raf/Erk- and Akt-pathways. Oncotarget. (2016) 7:55026–42. doi: 10.18632/ONCOTARGET.10392 PMC5342399 27391150

[46] Ichimura K Fukushima S Totoki Y Matsushita Y Otsuka A Tomiyama A. Recurrent neomorphic mutations of MTOR in central nervous system and testicular germ cell tumors may be targeted for therapy. Acta Neuropathologica. (2016) 131:889–901. doi: 10.1007/s00401-016-1557-x 26956871

[47] Tan JY Wijesinghe IVS Kamarudin MNA Parhar I. Paediatric gliomas: BRAF and histone H3 as biomarkers, therapy and perspective of liquid biopsies. Cancers. (2021) 13:1–18. doi: 10.3390/CANCERS13040607 PMC7913734 33557011

