# Supplementary material for: Plasma miRNA expression profile in pediatric pineal pure germinomas
Source: Front Oncol. 2024 Apr 11;14:1219796. doi: 10.3389/fonc.2024.1219796 (PMC11043570; doi:10.3389/fonc.2024.1219796)
Supplement: Supplementary file 1 [file DataSheet_1.docx]

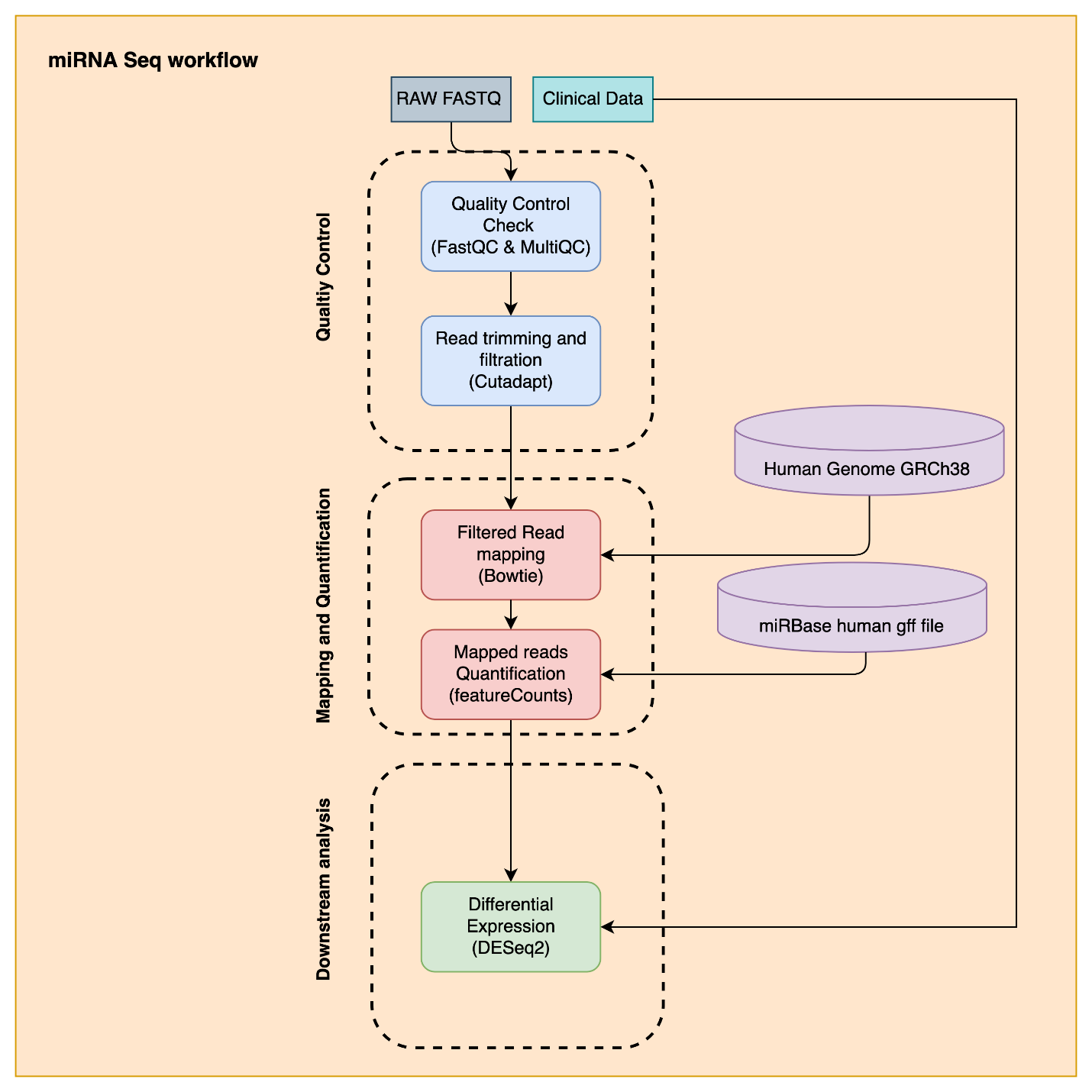


**Supplementary Figure 1**


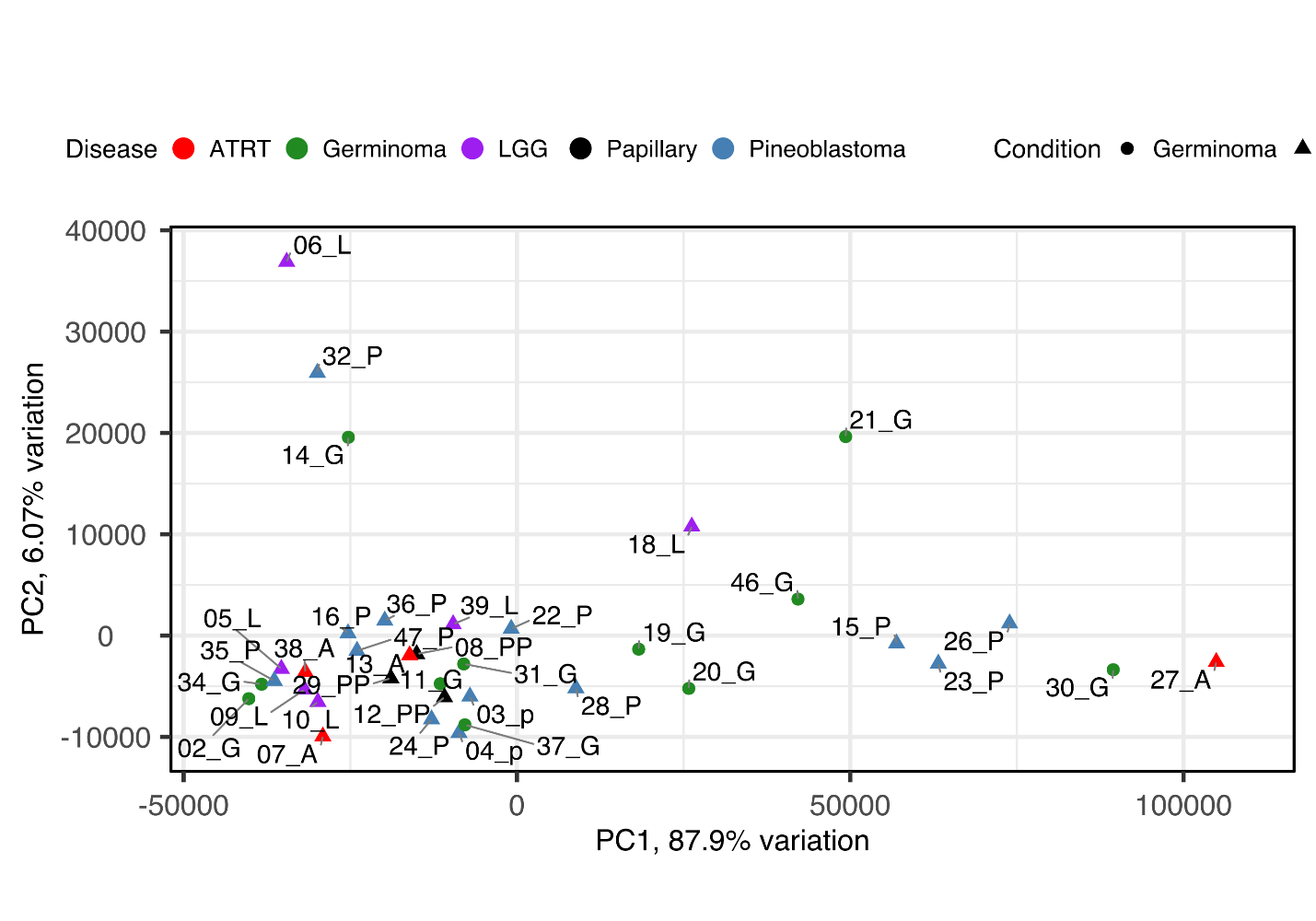


**Supplementary Figure 2**

**Supplementary Material**

**Supplementary Figure 1.** Summary of Bioinformatics pipeline.

**Supplementary Figure 2.** Principle component analysis (PCA) between all study samples where the point shape indicates the condition status (germinoma represented by circle) or other tumors (represented by triangle). Different diseases are represented with a specific color.
